# Rapid Implementation and Evaluation of Virtual Health Training in a Subspecialty Hospital in British Columbia, in Response to the COVID-19 Pandemic

**DOI:** 10.3389/fped.2021.638070

**Published:** 2021-05-19

**Authors:** Kasra Hassani, Theresa McElroy, Melissa Coop, Joelle Pellegrin, Wan Ling Wu, Rita D. Janke, L. Kit Johnson

**Affiliations:** ^1^Child Health British Columbia (BC), Provincial Health Services Authority, Vancouver, BC, Canada; ^2^Department of Occupational Science and Occupational Therapy, Faculty of Medicine, University of British Columbia, Vancouver, BC, Canada; ^3^British Columbia Children's Hospital Research Institute, Vancouver, BC, Canada

**Keywords:** implementation, evaluation, telehealth, training, capacity building, virtual health

## Abstract

**Introduction:** Adoption of virtual health (VH) solutions in healthcare has been challenging; this changed rapidly after implementation of physical distancing measures due to the COVID-19 pandemic. In response to the pandemic, British Columbia's Children's and Women's sub-specialty hospitals rapidly trained and scaled up support to equip staff and clinicians to use VH.

**Methods:** Ninety-minute live online training workshops and frequently updated online support materials were offered for 6 weeks. Training was monitored *via* feedback collected at training sessions and a brief post-training survey. After training completion, a second survey was circulated to measure utilization outcomes and experiences with VH.

**Results:** Eight hundred and ninety-five participants representing 82% of staff requiring support were trained through 101 sessions; 348 (38.9%) and 272 (30.4%) responses were collected for the monitoring and outcome surveys, respectively. Overall, 89% agreed that training was relevant to their needs; participants indicated average 58.1% (SD = 26.6) and 60.6% (SD = 25.2) increase in knowledge and confidence in VH after training; 90.1% had booked or conducted VH sessions. Increase in confidence was more pronounced in participants with lesser previous exposure to VH, but number of sessions conducted post-training and percentage of successful sessions were independent of previous exposure. For future training and support, participants suggested subject-tailored trainings, asynchronous trainings, and availability of experienced users.

**Discussion:** Training is key to success of VH implementation. Moving forward, core competencies in VH should be developed to support standardization and allow for evaluation and quality improvement. Incorporation of VH training in continuous professional development and onboarding is also highly recommended.

## Introduction

Despite a global movement toward digital technologies, adoption of virtual health (VH) solutions has been challenging and slow (1–4). This trend changed rapidly after the implementation of physical distancing measures due to the COVID-19 pandemic. VH became necessary for safe and timely patient care, and many barriers to its scale-up were overcome (5–8).

VH, also referred to as virtual care, telehealth, or telemedicine, is any non-face-to-face activity to deliver care. It encompasses both patient–provider and provider–provider encounters. The benefits of VH are especially pronounced during infectious disease outbreaks such as the COVID-19 pandemic, e.g., remote triaging, remote diagnosis, and consultations (7, 9, 10). However, the promise of VH includes opportunities such as (1) innovative health service delivery through virtual care technologies, e.g., virtual visits, digital messaging, remote or real-time monitoring; (2) providing care closer to home, e.g., local and regional health care teams, continuing education; and (3) increasing children's access to the output of research and technology. VH is considered a more patient-centered model, increasing access, offering comfort and convenience of being in the community, and reducing the cost and burden of travel to receive care (11, 12).

Implementing VH can pose numerous challenges. The health system's inertia toward new models of care, lack of technology infrastructure, regulatory and legal issues, lack of financial incentives, and low tech-literacy have historically slowed implementation of VH (2). Furthermore, despite recommendations for VH training and core competencies (13, 14), formal VH training programs are not widely established or studied (4, 9, 11, 15). For patients, lack of access to technology and connectivity, privacy and security concerns, and low tech-literacy hinder utilization of VH (11, 12, 16). Some of these challenges, such as reluctance and inertia, and to some extent financial incentives, have been overcome due to the necessity created by COVID-19; others remain, particularly addressing training and education needs (16).

In response to the COVID-19 pandemic and the British Columbia (BC) public health officer's call to stay at home, BC's Children's and Women's (C&W) hospitals rapidly implemented VH solutions and training across clinics and programs. Non-emergency patient visits ceased, while clinics rapidly trained and scaled up support to equip the staff and clinicians to use VH. This paper explores the development, implementation, and evaluation of the training module designed to support staff to use VH and offers lessons learned on development and implementation of VH for healthcare providers.

## Methods

### Training Content

The live online training workshop included the following content: (1) introduction to VH, including definition, types, and advantages and disadvantages; (2) clinical requirements for conducting virtual sessions, such as confirming patient identity, ensuring privacy, appropriate etiquette, and documentation; (3) the operational procedures for scheduling a VH visit, including collecting informed consent before the visit; (4) equipment required and available, and how to test before a visit; (5) an introduction to the two VH platforms Skype for Business and Zoom for Healthcare, including how to schedule a visit, how to use the software platforms on desktop and mobile devices, and how to troubleshoot common audio and video issues during a visit. Training slides and online resource documents were available to the participants before the training. Content was updated during implementation as per feedback by participants, input from collaborators, or evolving context (e.g., software updates, new operational procedures).

### Train-the-Trainer Model

Fifteen Child Health BC staff members, including 10 trainers, were redeployed from their primary roles and were trained to facilitate the live-online modules by the Child Health BC Manager of VH. Redeployed Child Health BC staff included provincial leads, research associates, and program coordinators and managers. All redeployed staff had 1–5 years previous experience in using VH platforms, although not necessarily for VH visits. Trainers practiced offering the training to one another. Those who joined the team later were trained by shadowing the live sessions followed by practice, and all had access to a training module lesson plan.

### Training Implementation

The training initiative's format was (1) 90-min live-online training workshops including question-and-answer sessions and post-session follow-up when required, and (2) online support materials such as Frequently Asked Questions (FAQs) and How To's for various topics and audiences.

Training was delivered *via* Skype for Business. Several sessions were scheduled for each day. Each session had a maximum class size of 12, later increased to 18, to encourage opportunity for interaction and ensure that support could be provided. Participants needed to take the training session only once. Each session included a lead trainer who delivered the content, a technical support trainer who assisted participants with technical issues and monitored the chat box, and a scheduled on-call trainer who would step in in case of technical difficulties or sudden change in the schedule of one of the trainers.

The project was managed through an Agile approach (17) and the training team met daily to discuss progress, logistics, and to incorporate the recently collected feedback into the training content.

### Recruitment

All C&W staff and clinicians who needed VH to continue patient care were encouraged to participate in the training; this included but was not limited to booking clerks, physicians, nurses, allied health staff, and nursing and administrative leads. Participants were invited to register *via* emails and reminders from their group leads and institutional communications.

### Monitoring and Evaluation

#### Monitoring

Training quality was monitored through informal feedback collected from participants by trainers during the session and a short post-training survey administered to participants through REDCap (18). Feedback collected during the session included suggestions for improvement in terms of training scheduling, content, and delivery, and questions not already addressed in the training. The post-training survey included two Likert-scale statements “The training was RELEVANT to my learning needs” and “I have the KNOWLEDGE and SKILLS to be successful in supporting or conducting a virtual health visit,” followed by two open-ended questions “What can we do to improve the training?” and “Please tell us of any additional support you need to support or conduct virtual health visits.” Data collection took place from March 27 to May 8, 2020 inclusive and feedback was added to a master list to provide project coordinators with quick access. The training leadership team reviewed the feedback weekly and incorporated the needed changes. Urgent feedback was raised and discussed at daily team meetings.

#### Outcome Evaluation

Two weeks after training program completion, a follow-up REDCap survey was sent to all participants. Focusing on short-term outcomes after the training initiative, the survey asked about changes in knowledge and confidence in VH, frequency of engagement in VH activities since the training, barriers and facilitators of conducting virtual sessions, and perspectives for the future. Specifically, participants were asked “How much did your SKILLS/CONFIDENCE for utilizing Virtual Health for patient visits increase following the training?” and responded using a visual analog scale (VAS) ranging from 0 to 100 with response anchors including not at all (0), moderately (50), and greatly (100). The survey remained open for 2 weeks and one reminder was sent *via* email after the first week.

### Data Analysis

Both quantitative and qualitative data were collected and analyzed. Quantitative data included responses to multiple-choice and VAS survey questions as well as overall administrative data. Quantitative data were analyzed using basic descriptive and inferential statistics in RStudio (19). Qualitative data included responses to open-ended questions in the monitoring and outcome surveys. To analyze the qualitative data, thematic analysis was applied to the dataset manually.

### Privacy Statement

As their primary purpose was monitoring and evaluation of an ongoing initiative, the study was exempted from Research Ethics Board review. Both surveys were reviewed and approved by the Provincial Health Services Authority Privacy Office.

## Results

### Live-Online Training

The training was live for 6 weeks. During this time, 10 trainers trained 895 participants through 101 training sessions; this represented 82% of C&W staff who required a VH solution to maintain care for patients. Class size varied between 1 and 20 (average = 8.8, SD = 4.5) with 2–6 daily sessions provided on weekdays.

### Supporting Materials

The project team created a landing page on the Child Health BC website to consolidate resources for learners, which allowed one-stop access to resources.

Eighteen supporting documents were produced and uploaded to Child Health BC website categorized by platform and audience. Overall, the page was viewed 1,049 times over the training period and the documents were downloaded 544 times.

### Monitoring

A total of 348 responses to the monitoring survey were collected throughout the training (38.9% response rate). Overall, 89.0% of the participants agreed or strongly agreed that the training was relevant to their learning needs and 84.4% indicated they had the knowledge and skills to successfully support or conduct a VH visit.

When possible, feedback was integrated in real time, e.g., updating content and FAQs for both platforms. Other feedback, such as the request for recorded sessions, was addressed over time or referred to partner organizations.

### Results of the Short-Term Outcomes Survey

#### Demographics

A total of 272 responses were collected for the follow-up survey (response rate 30.4%). The participants came from diverse clinics on the C&W campus and included a range of roles, such as nursing leadership, direct care staff, allied health members, and physicians. The majority (75.5%) had used VH zero to five times in the year before training, and 40.6% had no previous experience. Less than 10% had previously used VH frequently (21 times or more).

#### Changes in Knowledge and Confidence in Using VH

**Figure 1:**
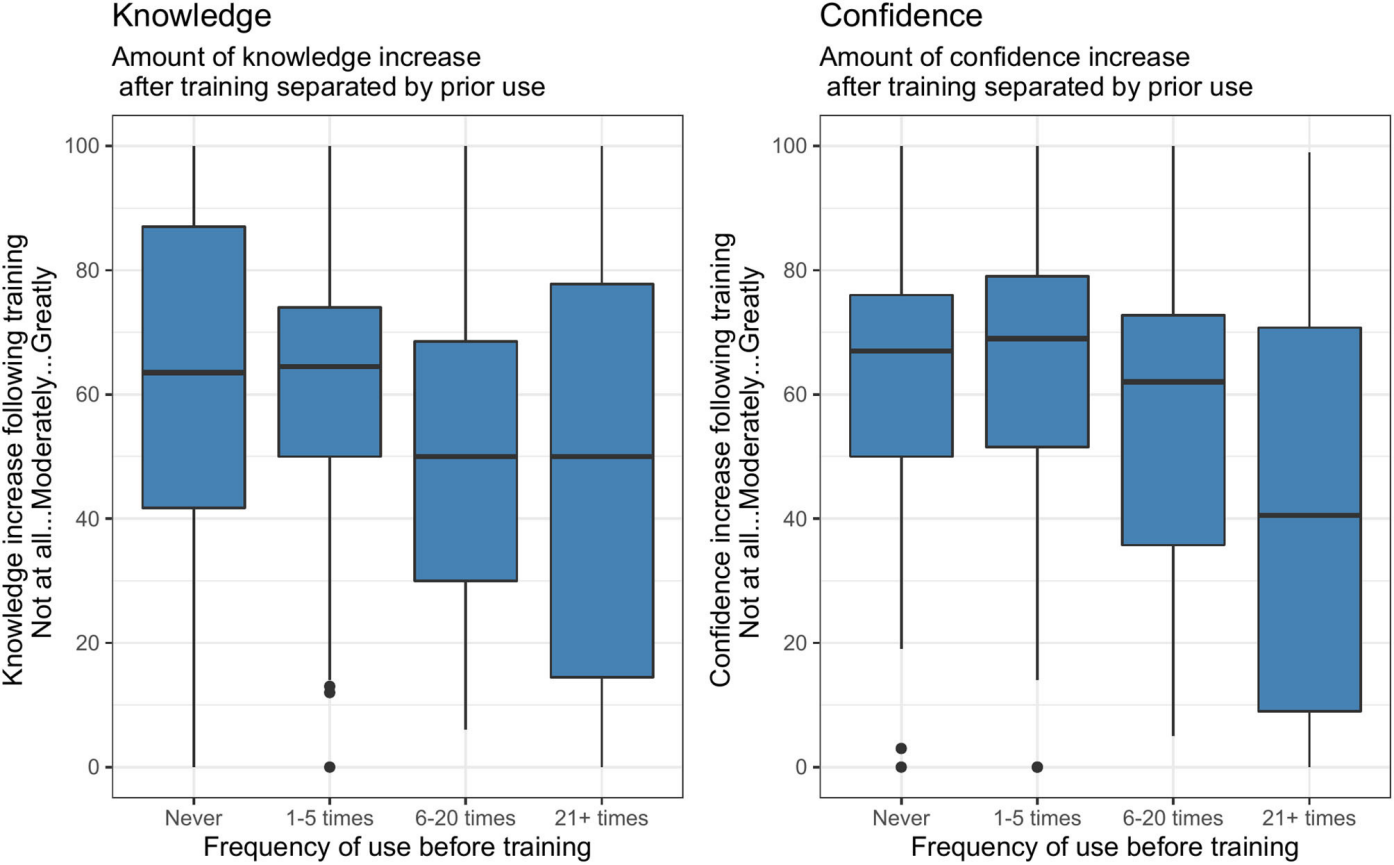
Self-assessed changes in knowledge and confidence post-training, separated by frequency of use in the year before. Changes in knowledge and confidence were more pronounced in those who had had lesser exposure to VH.

Overall, the participants self-reported an average increase of 58.1% (SD = 26.6) in knowledge and 60.6% (SD = 25.2) in confidence after the training. Figure 1 shows the self-assessed changes in knowledge and confidence for setting up and conducting VH sessions, separated by their previous use of VH in the year before the training. As can be seen in the boxplots, the increase was higher for participants with lesser previous exposure. This was more pronounced for confidence, compared with knowledge. Further analysis of the groups using one-way ANOVA shows that the difference in knowledge gained between groups was not statistically significant [*F*_(2,213)_ = 2.879, *p* = 0.058], but difference in confidence gained was significant [*F*_(3,217)_ = 5.738, *p* = 0.00373].

#### Training Usefulness and Future Directions

Overall, most participants found instructions on how to set up and conduct Zoom meetings helpful (75%), followed by clinical requirements for conducting VH sessions (44.5%). Suggestions for future training mirrored findings of the post-training monitoring survey, with top suggestions including trainings tailored by subject (e.g., specific to Zoom, Skype for Business, or other foci like clinical requirements and procedures, booking, and how to use breakout rooms), trainings offered through differing learning modalities, identified super users who could provide continuous support, and live hands-on demonstrations. This was followed closely by asynchronous learning options, such as videos and online courses. In addition, when asked what support was helpful following the trainings, a number of participants mentioned in-person support and access to educational materials.

#### How the Participants had Used VH After the Training

In response to whether they had used VH in the past few weeks since the training, over 90% of the participants responded positively. This included booking (38.6%) or participating (72.8%) in virtual team meetings, and booking (38.6%) or conducting VH sessions (52.9%). Reasons for not using VH (9.9%) included not having technology or programs set up in clinic, or not being applicable (e.g., bedside nurse).

Among those who had used VH, Zoom was the most commonly used platform, followed by Skype for Business. Other platforms or systems used included telehealth, telephone, Microsoft Teams, doxy.me, and Blue Jeans.

Of the participants who had conducted VH sessions, the majority mentioned that most to all of their sessions were successful (Table 1). This was independent of the participants' previous exposure to VH (Fisher's exact test: *p* = 0.8563). Success was defined in the survey as the clinical goals of the session being achieved.

**Table 1:** Estimated percentage of successful VH sessions for participants with different previous exposures to VH.

	**Estimated percentage of successful VH sessions**			
**Frequency of using VH before training**	**0–40%**	**41–80%**	**81–100%**	**Not applicable**
0–5 times	5 (2.8%)	48 (27.1%)	106 (59.9%)	18 (10.2%)
6–20 times	2 (4.7%)	15 (34.9%)	22 (51.2%)	4 (9.3%)
21+ times	0 (0%)	6 (27.3%)	13 (59.1%)	3 (13.6%)
**Overall**	**7 (2.9%)**	**69 (28.5%)**	**141 (58.3%)**	**25 (10.3%)**

*Overall, participants mentioned that the majority of their sessions were successful. There was no statistically significant difference between the groups (Fisher's exact test: p = 0.8563, alternative hypothesis: two-sided)*.

Majority (57.9%) of participants who had used VH post-training had booked or conducted between 10 and 100 sessions (Table 2). A smaller percentage (4.1%), including mostly participants who had taken the earlier training, had booked or conducted over 100. The interval between the training and the survey was between 2 and 8 weeks, depending on when the participants had taken the training. This therefore translates to 1–10+ weekly sessions. Previous exposure to VH did not have a significant relationship with number of sessions booked post-training (Fisher's exact test: *p* = 0.1606).

**Table 2:** Estimated number of sessions booked or conducted in the weeks following the training.

	**Estimated number of sessions conducted or booked since the training**			
**Frequency of using VH before training**	**0–9**	**10–100**	**100+**	**Many, I don't know**
1–5 times	50 (34.5%)	87 (60%)	5 (3.4%)	3 (2.1%)
6–20 times	11 (32.4%)	17 (50%)	2 (5.9%)	4 (11.8%)
21+ times	5 (33.3%)	8 (53.3%)	1 (6.7%)	1 (6.7%)
**Overall**	**66 (33.8%)**	**112 (57.9%)**	**8 (4.1%)**	**8 (4.1%)**

*Previous exposure to VH did not have a significant relationship with number of sessions booked post-training (Fisher's exact test: p = 0.1606, alternative hypothesis: two-sided)*.

#### Success of the Sessions: Barriers and Facilitators

**Figure 2:**
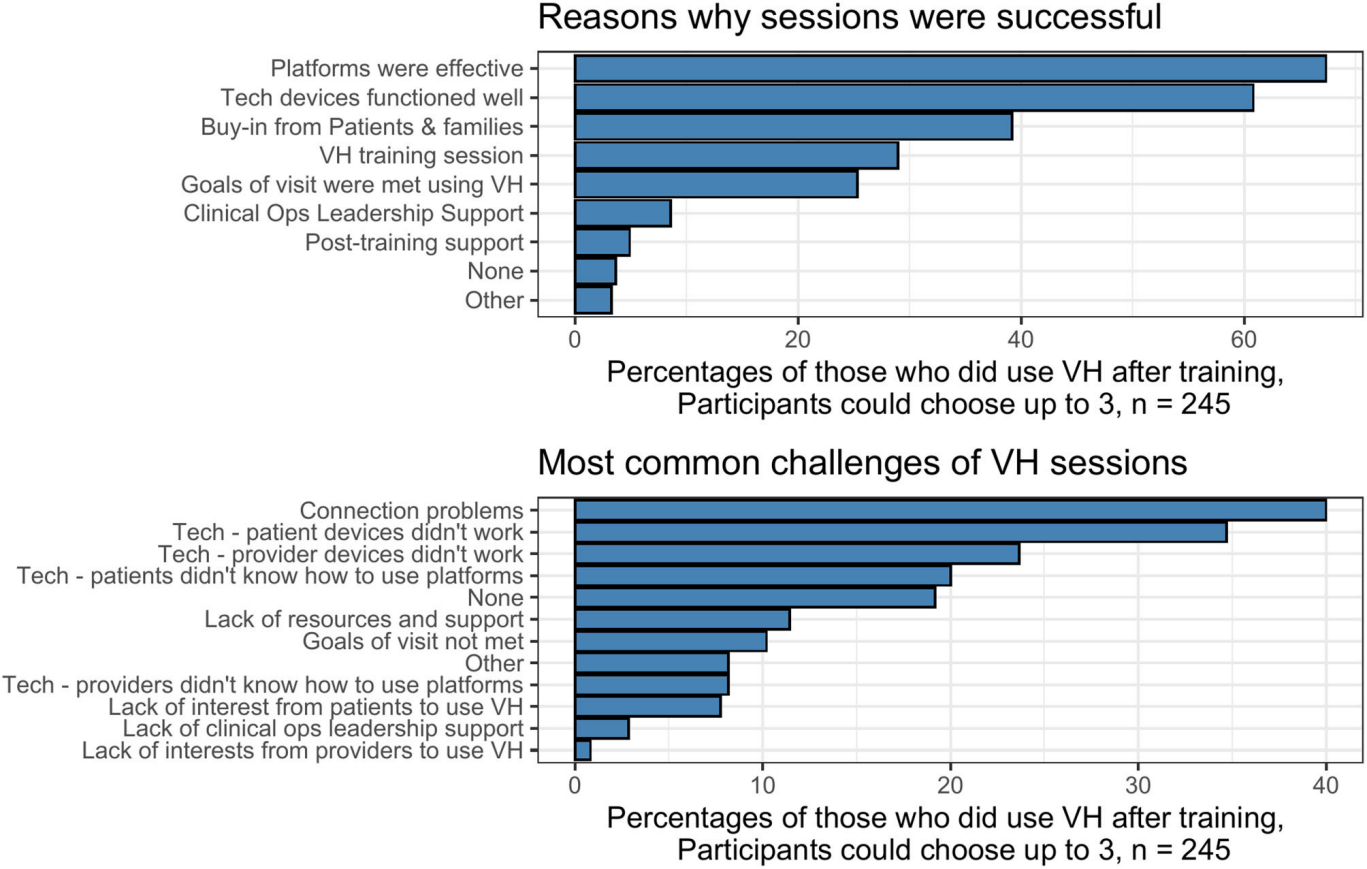
Facilitators and barriers to the success of the VH sessions, as indicated by the participants.

Most common facilitators and barriers to the success of the VH sessions are shown in Figure 2. Effective platforms, functioning devices, and buy-in from patients were the choices most commonly selected as reasons for success.

“*Families were more available via video call than to come here from, for example, [remote town]. We reached families that we normally would not.”*—Nurse

Connection and technology problems were also the main challenges of the sessions. A number of participants also mentioned not having access to equipment, e.g., headphones.

“*Not having phone/computer speakers available in all the clinic rooms.”*—Nurse

A number of practitioners mentioned patient behavioral challenges, which made the session less effective.

“*Client walking around, not staying in view, children sitting in front of camera even though it was an appointment to interview parents, no one wearing headsets in the house so tinny sounding sound… made for long and frustrating 90-minute experiences for me and added no value to my clinical work.”—*Physician

#### Using VH in the Coming Six Months

Over half of the participants indicated that they expect to regularly use VH in the next six months. This was the case for all participants, regardless of how often they had used VH before (Fisher's exact test: *p* = 0.1474).

“*It has been very positive for families and me. I think the training provided a good foundation. After that you just have to do it and learn as you go.”*—Physician

## Discussion

Necessity is a powerful change agent. While the benefits of VH were known before the COVID-19 pandemic (11, 12), the pandemic prompted a rapid shift to VH that would have been highly unlikely in typical circumstances (5, 6, 16). In our context of a Children's and Women's health center, more than half of participants surveyed indicated they would use VH regularly in the next six months despite limited to no exposure to VH before the pandemic. VH became a necessity for the continuation of care.

Lack of provider and staff education has been a key barrier to large-scale adoption of VH (4, 9, 11, 15), and it needed to be addressed rapidly; therefore, our team developed and implemented a training program to support a new virtual model of care. Through its evaluation, opportunities were identified to optimize VH training moving forward. Training options tailored to user needs was a notable theme in the evaluation and should be factored in during curriculum development for continuing professional development (CPD). While we were unable to provide tailored training in the rapid implementation necessitated by the pandemic, it is a promising strategy to maximize efficiency and outcomes of training in time-restrained clinical contexts. The recommendations included platform-specific learning (e.g., Skype for Business vs. Zoom for Healthcare), function-focused (booking vs. clinical use), and skill level (entry vs. follow-up for experienced practitioners). It was also suggested that diverse approaches to training be offered—both synchronous (e.g., webinars, hands-on training with superusers) and asynchronous (e.g., videos, handouts, FAQs) to address differing needs, learning styles, and time availability. Embedding these trainings into the workplace and garnering leadership to support the necessity of CPD in VH were important factors for the high rates of training completion, which should be considered by planners.

One of the significant and immediate outcomes of training was the increase in confidence, a finding also noted by others (11, 20, 21). As might be expected, this was more pronounced with participants who had no previous exposure to VH. However, many indicated that despite gains in knowledge and confidence, they needed further practice. The pandemic context meant that the majority of the participants booked or conducted VH sessions in the relatively short period after the training. Knowledge and confidence were solidified by inevitably practicing through sessions, using the platforms, technical trouble-shooting, and in-house support. This practice has been shown to be important for developing specific skills in VH (4, 20, 22). This learning through direct experience in clinical sessions was perhaps more acceptable in the context of the pandemic, where it was widely acknowledged that everyone was navigating new territory to the best of their ability. Moving forward, building hands-on practice opportunities into CPD is highly recommended.

Most VH sessions post-training were rated as successful. The most prominent facilitators related to success included effectiveness of platforms, devices that functioned well, and buy-in from patients and families. Challenges were most often related to connectivity and technology, either on the provider or the patient end, matters that were beyond control of the parties. These findings regarding barriers and facilitators are not new (11, 12) but compared with former studies, there was a notable shift: fewer structural barriers such as access to platforms, security, leadership support, and reimbursement were noted. This is likely because these barriers were being addressed with an unprecedented speed by leadership and technical teams, a finding noted by other teams who implemented VH during the pandemic (6). Further, there were fewer attitudinal or provider-specific barriers (12); the pandemic context pushed acceptance as there was a wide practical recognition that VH was now necessary for patient care to safely continue.

This Quality Improvement (QI)–focused evaluation highlighted the importance of monitoring and evaluation of the transition to VH. Barriers were quickly identified and were addressed when possible. Future evaluation should include more objective measurements of training effectiveness and monitoring VH usage trends and “success” rates. Success of VH sessions requires definition. In our survey, we used “goals of the session were met;” this definition could become more elaborate to capture different dimensions of a VH visit, such as clinical goals, technological issues, communication, and importantly patient perspectives. Documentation of failed sessions provides foundation for QI. There is also a need for more research focused on effective approaches to VH training and education (4, 9).

Offering more support for patients and families was another important theme; this could include more accessible equipment (e.g., loan programs), working with stakeholders to improve connectivity for remote or vulnerable families, and training and support (e.g., phone-in support line) (16). Patient support resources have been shown to decrease preparation burden for practitioners (11, 13, 14). As part of core competencies, VH training should include how to familiarize patients with VH technology, such as through basic guidelines or checklists, and in addition, techniques to empower patients.

Future training should incorporate core competencies to standardize care delivery through VH and allow for QI (4, 8, 9, 11, 13–15). Such core competencies have been developed in fields such as nursing (8, 13), emergency medicine (14), and behavioral health (23). Core competencies were not covered in detail in our training due to the rapid nature of the project, but should be included in future work. Others have also pointed out the need for curriculum development on regulation of VH, such as policies, procedures, protocols, etiquette, and ethics (8, 13, 15, 22). It is recommended that these curricula should be based on existing competency-based outcome-oriented frameworks such as CanMEDS (24). We also need to consider how to effectively assess VH competency in staff (25), and then provide tailored education and support as part of onboarding and maintenance. This ensures that practice continues to develop and evolve alongside our rapidly changing world.

## Limitations

Data used in this evaluation were from cross-sectional self-report surveys, and therefore are subject to common biases of survey data, such as response bias and confirmation bias. We believe that due to the relatively high response rate (30%), the risk of non-response bias is low. The data are a snapshot in time and have not measured any across-time changes and trends. Finally, the results have not been substantiated with objective data such as number of VH sessions booked and the input from patients and families has not been captured. We aim to address these measurement limitations in future work.

## Conclusion

The initiative was successful in rapidly preparing staff and providers to provide VH at the onset of the COVID-19 pandemic, where VH use became a necessity. Next steps should include focus on the development of core competencies, diversifying training modalities, incorporation of VH education into onboarding and continuous professional development, and rigorous evaluation.

## Data Availability Statement

The raw data supporting the conclusions of this article will be made available by the authors, without undue reservation.

## Ethics Statement

Ethical review and approval was not required for the study on human participants in accordance with the local legislation and institutional requirements. The patients/participants provided their written informed consent to participate in this study.

## Author Contributions

KH, TM, and RJ designed the evaluation. KH and TM conducted the monitoring evaluation, analyzed data, and wrote the article. JP, MC, and KJ developed the training. JP, MC, and WW led the training and the training team. All co-authors contributed to the results interpretation and the discussion.

## Conflict of Interest

The authors declare that the research was conducted in the absence of any commercial or financial relationships that could be construed as a potential conflict of interest.
